# Blockchain and Internet of Medical Things for Resilient Healthcare

**DOI:** 10.3390/bioengineering13101173

**Published:** 2026-10-08

**Authors:** Graziella Di Grezia, Alfredo Clemente, Stefano Dugheri, Vincenzo Mattei, Alessio Gili, Emanuela Mari, Fabrizio Urraro, Nicola Mucci, Antonio Baldassarre

**Affiliations:** 1 Department of Life Science, Health, and Health Professions, Link Campus University, 00165 Rome, Italy; g.digrezia@unilink.it (G.D.G.); s.dugheri@unilink.it (S.D.);; 2 RADIOCHAIN S.r.l., Academic Spin-Off of the University of Campania “Luigi Vanvitelli”, 80138 Naples, Italy; 3 Department of Experimental and Clinical Medicine, University of Florence, 50134 Florence, Italy

**Keywords:** blockchain, Internet of Medical Things, IoMT, IoT, digital health, cyber-physical systems, patient-centric healthcare, electronic health records, federated learning, homomorphic encryption, AI Act

## Abstract

The COVID-19 pandemic exposed persistent weaknesses in health-data interoperability, cybersecurity, provenance, cross-organizational governance, and continuity of digital care. This short communication presents an updated focused narrative synthesis and a five-layer governance framework for integrating blockchain and the Internet of Medical Things (IoMT) in patient-centered digital health. The literature was updated through 10 August 2026, with recent peer-reviewed evidence complemented by authoritative standards and European regulatory sources. The synthesis positions blockchain primarily as a distributed governance, provenance, and audit layer rather than a clinical database or stand-alone security solution, while IoMT provides heterogeneous sensing infrastructure for continuous and distributed care. The framework separates: (1) device and data acquisition; (2) network and edge orchestration, including 5G/SDN where clinically justified; (3) blockchain-based governance; (4) privacy-enhancing data, analytics, and AI; and (5) application and service delivery. Homomorphic encryption, zero-knowledge proofs, secure aggregation, differential privacy, and federated learning are treated as complementary controls with distinct threat models and computational trade-offs. The revised framework also incorporates the European Health Data Space, the EU AI Act and its medical-device interplay, and NIS2 as a regulatory overlay. Across six use-case domains, evidence is strongest for technical feasibility, provenance, and programmable access control; evidence for clinical effectiveness, cost-effectiveness, equity, sustainability, and large-scale deployment remains limited. The proposed model therefore emphasizes standards-based interoperability, minimization of on-chain personal data, endpoint trust, auditable human oversight, and prospective real-world validation.

## 1. Introduction

The COVID-19 pandemic acted as an unprecedented stress test for health-system digital infrastructure, accelerating remote care, cross-institutional data exchange, and population-level surveillance while exposing persistent weaknesses in interoperability, cybersecurity, provenance, and governance [1,2]. These weaknesses were heterogeneous across settings and cannot be attributed to centralization alone; rather, they reflected fragmented information architectures, uneven standards implementation, rapidly changing workflows, and limited mechanisms for accountable data sharing across organizational boundaries.

The consequences were clinical and organizational as well as technical. The pandemic highlighted the importance of timely access to longitudinal records, trustworthy public-health data, resilient pharmaceutical supply chains, and accountable use of rapidly deployed digital and AI-enabled tools [2,3,4]. It therefore strengthened the case for architectures that combine interoperability standards with explicit security, provenance, consent, accountability, and continuity-of-service mechanisms rather than treating these requirements as interchangeable properties of a single technology.

Two technology families are particularly relevant to this problem space: distributed-ledger technologies, including blockchain, and the Internet of Medical Things (IoMT). The IoMT, the healthcare-specific extension of the Internet of Things (IoT) [5,6], encompasses wearable biosensors, implantable devices, connected therapeutic equipment, point-of-care sensors, and other networked devices capable of generating continuous or episodic health data [7,8]. 5G, edge computing, and Software-Defined Networking (SDN) can improve latency, traffic orchestration, and policy enforcement for selected remote-monitoring scenarios, although their necessity and performance benefits depend on the clinical use case and deployment context [9]. Recent empirical work continues to explore blockchain–IoMT architectures for secure EHR and monitoring workflows [10,11].

Blockchain, initially developed as distributed trust infrastructure for cryptocurrency, has evolved into a broader class of programmable distributed ledgers [12,13,14]. Its useful healthcare properties include tamper-evident append-only records, distributed validation, cryptographic provenance anchors, and programmable smart contracts. These properties can support governance where multiple organizations need a shared audit and coordination layer, but they do not by themselves ensure confidentiality, semantic interoperability, data quality, or legal compliance [15,16,17]. Hybrid architectures therefore generally keep high-volume or directly identifiable clinical content off-chain while placing hashes, access events, consent-policy state, or provenance metadata on-chain; dual-ledger models are one possible refinement rather than a universally preferred architecture [18,19,20].

Healthcare Cyber-Physical Systems (HCPS) provide a useful systems perspective by integrating physical sensing, computation, communication, and feedback loops [21]. Within such systems, patient-centric software agents can mediate access to IoMT streams according to encoded policies and generate auditable delegation events [9]. Privacy-enhancing technologies, including homomorphic encryption, zero-knowledge proofs (ZKPs), differential privacy, secure aggregation, and federated learning, can complement rather than replace conventional cybersecurity controls [22,23,24]. Likewise, machine-learning-based intrusion detection may help identify anomalous network behaviour, but blockchain cannot establish the truthfulness of a compromised sensor reading before that reading is anchored to the ledger [25].

The Industry 5.0 paradigm provides a useful ethical and design lens through its emphasis on human-centricity, sustainability, and resilience [26,27,28]. In Europe, this design agenda now intersects with a rapidly evolving regulatory environment for health-data access and reuse, AI governance, medical-device software, and cybersecurity, including the European Health Data Space (EHDS), the EU Artificial Intelligence Act (AI Act), its 2026 amendment, and the NIS2 framework [29,30,31,32,33]. The framework proposed below therefore treats blockchain as one governance component within a wider socio-technical and regulatory architecture.

## 2. Materials and Methods

### 2.1. Search Strategy and Source Integration

This communication is based on a focused narrative review of peer-reviewed literature originally spanning 2017–2026 across PubMed/MEDLINE (National Library of Medicine, 8600 Rockville Pike, Bethesda, MD 20894, USA), IEEE (Institute of Electrical and Electronics Engineers, 3 Park Avenue, New York City, NY, USA) Xplore, Scopus (Elsevier B.V., Amsterdam, The Netherlands), and Google Scholar (Googleplex, Mountain View, CA, USA). For the present revision, targeted update searches were conducted through August 2026 to identify relevant 2017–2026 peer-reviewed studies and authoritative standards, guidance, and regulatory documents. The literature was organized using the following Boolean search clusters:

- “blockchain AND healthcare” OR “distributed ledger AND electronic health record”;
- “Internet of Medical Things” OR “IoMT AND clinical workflow” OR “IoT AND patient monitoring”;
- “5G AND healthcare IoT” OR “SDN AND remote patient monitoring”;
- “dual blockchain AND health” OR “off-chain storage AND EHR”;
- “homomorphic encryption AND healthcare” OR “federated learning AND health data”;
- “patient-centric agent AND IoMT” OR “smart contract AND consent management”;
- “Industry 5.0 AND healthcare” OR “COVID-19 AND digital health governance”.

Synthesized findings were organized across thematic clusters covering patient-centricity and data agency; security and privacy; supply-chain resilience; interoperability; clinical deployment; scalability and performance; energy and sustainability; and regulatory governance. Targeted update searches additionally used combinations of “European Health Data Space”, “AI Act”, “medical device AI”, “NIS2”, and “FHIR” with healthcare, IoMT, or blockchain terms.

The search strategy followed a stepped approach. Initial database queries across the five sources (PubMed/MEDLINE, IEEE Xplore, Scopus, Google Scholar) returned 1247 records before duplicate removal, consistent with the scale reported in recent systematic reviews in this domain. After title and abstract screening, 178 full-text articles were assessed against the inclusion criteria; 127 were retained for thematic synthesis. An additional 37 regulatory, standards, and guidance documents were incorporated to update the governance framework, including the European Health Data Space, the AI Act and its 2026 amendment, the AIB/MDCG interplay guidance, and the NIS2 Directive. This selection process reflects a narrative review methodology rather than a systematic review: unlike a systematic review, which follows a pre-registered protocol, a fixed PICO-type question, and standardized guidance such as the Cochrane Handbook or the PRISMA statement, a narrative review has no equivalent mandatory protocol and instead allows the broader, cross-disciplinary synthesis needed for a fast-evolving, multi-domain topic spanning technology, cybersecurity, clinical workflow, and evolving European regulation [34,35]. The review was accordingly not prospectively registered in a protocol repository such as PROSPERO, and the coverage of peer-reviewed literature through August 2026 aligns with the temporal scope of recent systematic updates in blockchain and federated learning for healthcare.

**Figure 1:**
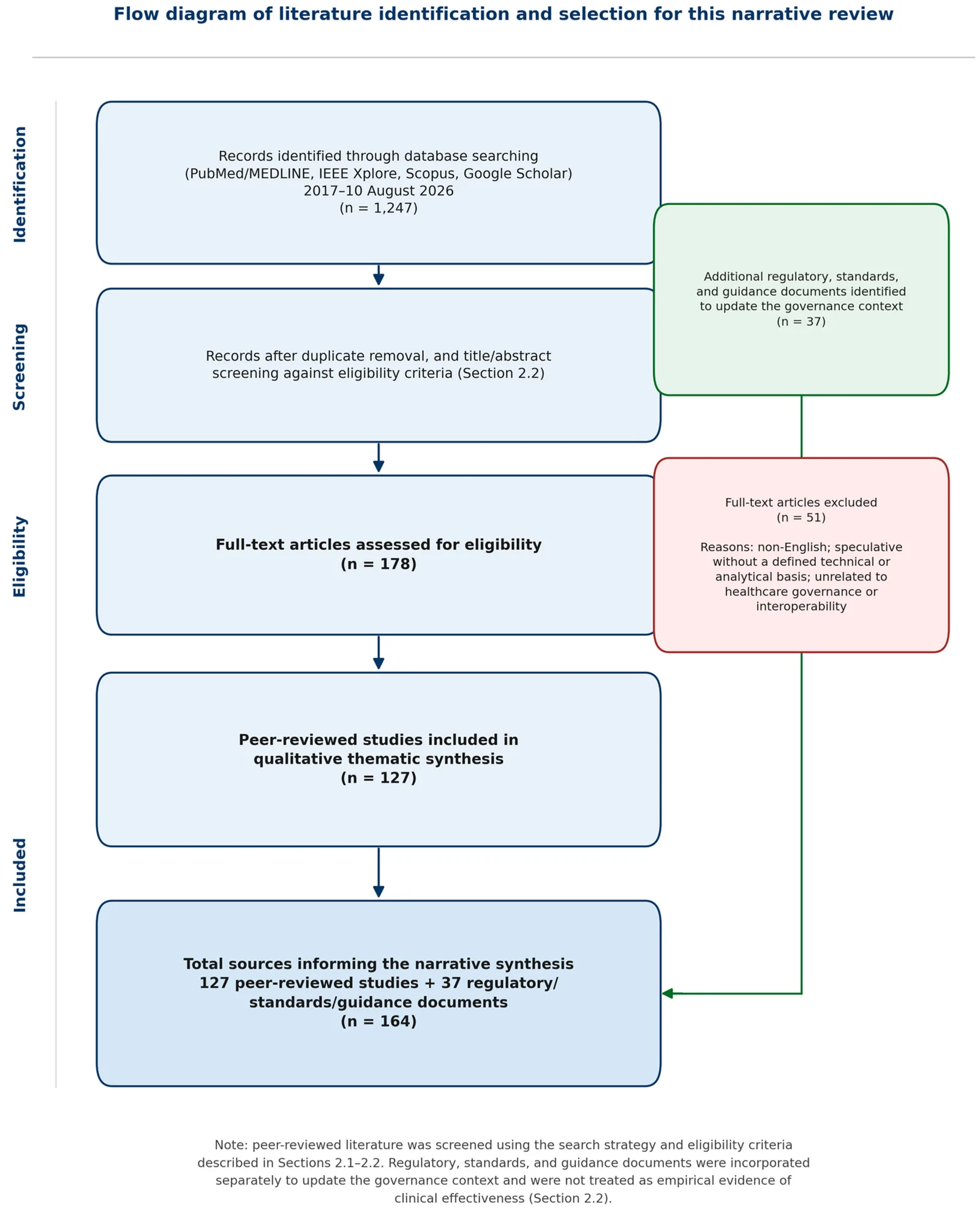
Flow diagram of literature identification and selection for this narrative review. Peer-reviewed literature was screened using the four-database search and eligibility criteria described; regulatory, standards, and guidance documents were incorporated separately and were not treated as empirical evidence of clinical effectiveness. This diagram is provided for transparency and does not indicate that the review followed a formal systematic review protocol.

To improve reproducibility, we clarify that each of the seven Boolean clusters above was applied, with minor syntax adaptations for each platform, across all four databases (PubMed/MEDLINE, IEEE Xplore, Scopus, Google Scholar), searching title, abstract, and keyword fields and restricting results to English-language, peer-reviewed items published between 2017 and 10 August 2026. A flow diagram summarizing record identification (*n* = 1247), duplicate removal, title/abstract screening, full-text assessment (*n* = 178), and final inclusion (*n* = 127 peer-reviewed studies plus 37 regulatory and standards documents) is provided below as Figure 1.

On terminology, we retain the designation “focused narrative review” throughout rather than “systematic review”. The two designs differ in objective, methodology, and application rather than merely in the size of the search: a systematic review formulates a narrowly defined, PICO-type question and follows a pre-registered, guideline-driven protocol (e.g., the Cochrane Handbook or PRISMA) with critical appraisal of every included study, typically by two independent reviewers, in order to support a single, well-bounded evidence synthesis; a narrative review has no equivalent mandatory protocol, may address a broader or multi-part question, and is better suited to tracking the development of a fast-moving, cross-disciplinary field, appraising previous work, and identifying knowledge gaps [34,35]. This manuscript follows the narrative form: study selection and evidence grading were carried out by the author team without a registered protocol, a fixed PICO question, or full-text critical appraisal performed independently by two reviewers throughout, so “systematic review” would overstate the methodological rigour applied. At the same time, the four-database search with explicit fields and dates, the numerically documented screening (1247 records to 127 included studies), the literature flow diagram, and the explicit evidence-grading rubric introduced in Section 3.5 go beyond typical narrative-review practice and are offered in the spirit of recommended quality standards for narrative reviews, such as the SANRA scale and established guidance on writing narrative reviews for peer-reviewed journals [34,35].

### 2.2. Inclusion and Exclusion Criteria

Peer-reviewed publications were included when they addressed at least one of the following in healthcare data governance or digital-health system design: blockchain architectures and consensus mechanisms; IoMT/IoT-enabled monitoring or clinical workflows; 5G, SDN, edge, or cyber-physical healthcare architectures; privacy-preserving computation; patient-centric access and consent mechanisms; Industry 5.0; interoperability; cybersecurity; or implementation barriers. Non-English publications, purely speculative pieces without a defined technical or analytical basis, and studies unrelated to healthcare governance or interoperability were excluded. Authoritative legal, regulatory, guidance, and standards documents were included separately to update the governance context; they were not treated as empirical evidence of clinical effectiveness.

### 2.3. Synthesis Approach

A qualitative thematic synthesis was conducted iteratively. Recurring themes were grouped around data provenance and audibility; on-chain/off-chain architectural patterns; IoMT connectivity and edge orchestration; patient-centric policy enforcement; privacy-enhancing computation; interoperability; implementation and regulatory constraints; and human-centric design. Evidence strength was graded qualitatively according to study design, independence of validation, realism of the evaluation environment, and whether the outcome concerned technical feasibility, operational performance, or clinical/organizational benefit. Prototype and simulation evidence was therefore not equated with demonstrated clinical effectiveness. These themes informed the five-layer framework, the use-case mapping, and the challenge-and-mitigation analysis.

## 3. Results

### 3.1. Blockchain Architectures: Platforms, Patterns, and Healthcare Fit

The literature describes three broad blockchain families relevant to healthcare: public permissionless networks, consortium or permissioned networks, and private permissioned ledgers [12,13,14,17]. Permissioned and consortium designs are frequently favored in healthcare prototypes because node participation, governance rules, identity management, and transaction visibility can be constrained to defined actors [15,16]. This architectural choice may facilitate data-governance controls, but it does not itself satisfy GDPR, HIPAA, EHDS, or sector-specific medical-device requirements. Hyperledger Fabric remains prominent in patient-centric and access-control prototypes because of permissioned membership, channel-based segregation, and programmable chain-code [36,37].

The choice of consensus mechanism within permissioned platforms has nontrivial implications for IoMT deployment, yet the literature provides limited head-to-head comparisons under realistic healthcare workloads. Hyperledger Fabric’s pluggable consensus architecture allows deployment with Raft (crash-fault-tolerant) or, in some implementations, Practical Byzantine Fault Tolerance (PBFT) variants; the selection affects transaction latency, throughput, and energy consumption. Liu and Li observed that permissioned consensus designs can achieve energy reductions compared to Proof-of-Work alternatives, but the specific energy savings depend on validator count, block interval, and transaction volume rather than on consensus family alone [38]. For IoMT edge scenarios, where device battery life and network bandwidth are constrained, lightweight consensus and batch aggregation have been proposed as architectural mitigations [39,40]. However, systematic evaluations of consensus performance under clinically realistic workloads, including peak telemetry rates, network partitions, and node failures, remain sparse; most prototype evaluations report performance under synthetic or low-load conditions.

Beyond platform selection, hybrid on-chain/off-chain storage is a recurrent architectural pattern. High-volume clinical content is generally retained in institutional repositories, object storage, databases, or content-addressed systems, while the ledger records cryptographic digests, pointers, authorizations, or provenance events [11,19,20]. Dual-blockchain or multi-ledger patterns can further separate governance domains, for example, patient-facing permissions from provider operational transactions [18]. However, no single combination should be regarded as a universal state of the art: suitability depends on threat model, data topology, latency requirements, governance model, recovery procedures, and the legal status of on-chain metadata.

### 3.2. IoMT and 5G/SDN: The Physical–Digital Interface

The Internet of Medical Things encompasses a device spectrum whose clinical and governance implications vary substantially. Across the integrated literature, IoMT devices relevant to post-pandemic governance cluster around four functional categories: body-worn and implantable sensors generating continuous personal physiological streams (wearable ECG, continuous glucose monitors, smart inhalers, cardiac implants); fixed clinical devices generating operational and compliance data (networked ventilators, infusion pumps, dialysis machines) [41]; ambient environmental monitors relevant to infection control and syndromic surveillance (air quality, hand-hygiene compliance, hospital capacity sensors); and logistics and supply-chain sensors ensuring pharmaceutical provenance (RFID/NFC tags, cold-chain temperature loggers, smart dispensing cabinets) [7,8].

A concrete instantiation of the on-chain/off-chain separation can be traced across several prototype architectures. Zhang et al. proposed a hierarchical data-sharing model in which patient data is stored off-chain in encrypted form, while attribute-based access control policies and data hashes are maintained on a permissioned blockchain; the hierarchy separates personal health records from device-generated IoMT telemetry to support differentiated access. Gohar et al. integrated blockchain, cloud, and IoT within a FHIR-compatible data model, using the blockchain for trust and policy coordination while retaining clinical content in cloud-based FHIR repositories. Othman and Getahun extended this pattern with a dual-layer architecture that separates patient identity management from clinical data exchange, demonstrating that ledger metadata can be minimized through careful schema design.

Across these architectures, a common data lifecycle emerges: (i) device-side acquisition and local validation, often with edge-based preprocessing to reduce transmission load; (ii) off-chain storage of clinical content in governed repositories (e.g., FHIR servers, object storage, or IPFS), with cryptographic hashes and storage pointers retained on-chain; (iii) smart-contract-mediated access control that evaluates patient consent, provider credentials, and purpose before granting data access; and (iv) auditable logging of all access events, policy changes, and delegation transactions on the ledger. This pattern addresses the volume and latency constraints that make direct on-chain storage of continuous IoMT telemetry impractical, while preserving the provenance and audibility that motivate blockchain adoption. However, as Zhuang et al. and Zhang et al. note, the security and privacy guarantees of this architecture depend on the integrity of the off-chain storage system, key-management practices, and the minimisation of re-identifiable metadata in on-chain transactions.

A relevant architectural development is the integration of programmable networking and edge computing with IoMT governance. Rahman et al. demonstrated a blockchain-enabled patient-centric agent operating through an SDN control plane in a 5G remote-monitoring prototype [9,42]. The model illustrates how network-layer orchestration can be coupled with policy-aware access control and auditable delegation. Its findings support technical feasibility under the tested conditions, not population-scale clinical effectiveness or universal privacy guarantees. Accordingly, the five-layer model treats 5G/SDN as a high-performance connectivity option within a broader network-and-edge layer rather than as a mandatory prerequisite for blockchain–IoMT governance.

### 3.3. Privacy-Preserving Computation: Homomorphic Encryption, ZKPs, and Federated Learning

The synthesis identifies complementary privacy-enhancing techniques that extend governance beyond access control into computation on sensitive data. Homomorphic encryption permits defined computations over ciphertext and can reduce plaintext exposure to an analytics provider; Ali et al. demonstrated this pattern in the HealthLock IoT-healthcare architecture [22]. The approach is attractive for cross-institutional analytics but remains computationally expensive for some workloads, and its use does not remove requirements for lawful processing, key management, minimization, or output-disclosure controls [24].

ZKPs can support selective verification without disclosing the underlying value, while federated learning enables distributed model training without centralizing raw institutional datasets. Blockchain may add model-version provenance, contribution attribution, coordination, or audit records to federated workflows [23,24,43]. These mechanisms are complementary rather than inherently privacy-preserving as a bundle: federated learning can still leak information through model updates, ledger metadata can create linkability risks, and ZKPs and homomorphic encryption introduce computational and implementation trade-offs. Recent reviews therefore emphasize secure aggregation, differential privacy, robust threat modelling, and careful coupling between the ledger and learning layers [23,24].

The operational implications of these privacy-enhancing techniques warrant closer examination. Homomorphic encryption, as demonstrated in the HealthLock architecture by Ali et al., enables encrypted computation over IoMT health data but introduces computational overhead that may limit deployment in real-time monitoring scenarios; the authors reported feasibility for batch analytics rather than continuous streaming. For federated learning, Wang et al. conducted a systematic review of blockchain-federated learning integration in medical contexts, identifying that while blockchain can provide model-version provenance, contribution attribution, and audibility, it does not inherently address privacy leakage from gradient updates or model inversion attacks; these require complementary mechanisms such as differential privacy and secure aggregation. The 2026 tutorial by Shahsavari et al. reinforces this conclusion, emphasizing that regulatory compliance must be engineered across the full data and model lifecycle rather than inferred from the presence of a privacy technology.

Recent architectures have extended this integration by combining blockchain-governed federated IoMT with reinforcement-learning-based consensus and resource forecasting, as demonstrated by Murthy and Shri, and by coupling blockchain, IoT devices, and federated learning in sustainability-oriented healthcare systems, as reported by Bhasker et al. These works illustrate a trend toward adaptive, context-aware privacy and resource management, but their validation remains predominantly simulation-based, with limited deployment in clinical production environments.

### 3.4. Patient-Centric Frameworks: From Access Control to Data Agency

A consistent theme is the shift from application-centric data control toward patient-centred governance and greater data agency. Recent architectures allow patients to hold or control credentials, view access histories, and express granular, time-limited, or revocable sharing preferences [36,37,44]. These mechanisms can improve transparency and participation but should not be equated with absolute legal “ownership” of health data, which varies by jurisdiction and processing context. Patient-centric agents and smart contracts can operationalize selected permission and delegation workflows for IoMT data [9].

Smart contracts can automate defined access-policy and consent-state transitions and can make those transitions auditable; they do not, however, confer GDPR, HIPAA, or EHDS compliance by themselves [18,29,44]. Likewise, semantic interoperability is supplied primarily by shared information models, terminologies, implementation guides, and governance agreements rather than by the ledger. Gohar et al. combine blockchain, cloud, IoT, and a FHIR-compatible data model, illustrating how a trust layer can complement standards-based interoperability [18]. The updated evidence supports feasibility and potential governance value, but it remains insufficient to conclude that blockchain–IoMT integration improves clinical outcomes across settings [11,16,17].

### 3.5. Synthesis: Framework Architecture and Evidence Map

Several prior reviews have proposed layered or reference architectures for blockchain-enabled IoMT, including systematic reviews of blockchain in healthcare and IoT, of blockchain-enhanced IoT ecosystems, and the 2025 systematic reviews of blockchain-based healthcare implementations [15,16,17,45,46]. The framework proposed here differs from these prior efforts in four respects: (i) it separates network/edge orchestration (5G/SDN) from blockchain governance as a distinct layer, treating high-performance connectivity as an optional rather than mandatory component; (ii) it embeds an explicit, auditable evidence-grading rubric (below; Table 4) rather than a narrative-only maturity assessment; (iii) it integrates the 2025–2026 European regulatory landscape, the EHDS Regulation, the AI Act as amended in 2026, and the AIB/MDCG interplay guidance as a fifth cross-cutting governance overlay rather than as a general discussion point; and (iv) it explicitly maps six use-case domains against calibrated evidence strength (Table 2) rather than presenting use cases as illustrative vignettes. The contribution is therefore integrative and governance-oriented rather than a proposal of new blockchain or IoMT technical primitives.

The resulting five-layer governance framework is presented in Table 1. It separates device acquisition, network/edge orchestration, distributed-ledger governance, privacy-preserving data and AI services, and application delivery so that responsibilities and failure modes remain explicit. Table 2 maps six use-case domains to the roles of IoMT and blockchain; Table 3 links key barriers to mitigation strategies; and Table 4 calibrates evidence strength across seven claims. Evidence ratings refer to the maturity and external validity of the supporting evidence, not merely the number of publications.

**Table 1:** Five-Layer Blockchain–IoMT Governance Framework for Digital Healthcare.

Layer & Scope	Key Components	Primary Governance Functions	Standards & Protocols
Layer 1 Device & Data Acquisition	IoMT wearables, smart implants, biosensors, connected medical devices, point-of-care sensors, ambient monitors, RFID/NFC supply-chain tags	Continuous physiological telemetry; device identity and authentication; structured data generation; provenance at acquisition; device-status and calibration metadata	HL7 FHIR R4/R5; IHE profiles; IEEE 11073; DICOM/DICOMweb; GS1 identification standards
Layer 2 5G/SDN Network & Edge	5G network slices, Software-Defined Networking (SDN) control planes, edge compute nodes, IoMT gateways, GPU inference clusters	Low-latency secure transmission where clinically required; traffic orchestration; segmentation and policy enforcement; edge preprocessing; local safety functions; federated-learning coordination	3GPP 5G; ETSI MEC; SDN/OpenFlow where used; TLS 1.3; OAuth 2.0/OpenID Connect
Layer 3 Blockchain Governance	Permissioned/consortium distributed ledger (e.g., Hyperledger Fabric or Besu), smart contracts, optional multi-ledger models, cryptographic hash anchors, decentralised identifiers and verifiable credentials	Provenance anchoring; consent-policy state and delegation; tamper-evident audit logs; cross-organisational identity and credential verification; policy orchestration; auditable break-glass access	W3C DID and Verifiable Credentials; platform-specific smart-contract frameworks; cryptographic hashes/content identifiers; organisational governance policies
Layer 4 Data, Analytics & AI	Governed off-chain repositories, federated-learning nodes, secure aggregation, homomorphic encryption, ZKPs, differential privacy, ML/AI decision-support platforms	Lifecycle-managed EHR/IoMT data; privacy-enhancing computation; model provenance and monitoring; AI-assisted decision support where validated; population-health analytics	HL7 FHIR; governed databases/object storage or IPFS where appropriate; ONNX; federated-learning frameworks; privacy-enhancing computation libraries
Layer 5 Application & Service Delivery	Clinician dashboards, patient portals and mobile apps, public health surveillance platforms, research repositories, regulatory reporting interfaces, telemedicine systems	Care coordination; patient-facing access and delegation; population-health monitoring; pharmacovigilance; research governance; regulatory and safety reporting	SMART on FHIR; DICOMweb; standards-based APIs; GDPR/EHDS governance controls; AI Act/MDR-IVDR and NIS2 requirements where applicable

**Table 2:** High-Value Healthcare Use Cases: IoMT and Blockchain Roles with Evidence Strength.

Use Case Domain	IoMT/IoT Role	Blockchain Role	Evidence Strength
Remote Patient Monitoring & Telemedicine	Wearables and biosensors transmit heart rate, BP, SpO2, and glucose in real time via 5G/SDN-enabled low-latency links to clinicians and AI triage systems	SDN-integrated patient-centric agent mediates access to IoMT streams via smart contracts; immutable access logs; DID-based patient identity across providers	Moderate—multiple prototypes and benchmarked architectures; limited clinical-scale validation (Rahman et al., 2024; Jabarulla & Lee, 2021; Othman & Getahun, 2025)
Pandemic & Emergency Response	Body-temperature arrays, SpO2 networks, ambient air-quality sensors for syndromic surveillance; resource-capacity sensors for hospital load monitoring	Tamper-evident contact-tracing logs; cross-jurisdictional outbreak data sharing with verified provenance; transparent public-health reporting	Moderate—extensive COVID-19 literature and selected deployments/pilots, but heterogeneous evidence and limited controlled outcome evaluation (Firouzi et al., 2021; Ng et al., 2021)
Pharmaceutical Supply Chain	RFID/NFC tags on drug packaging; cold-chain temperature sensors; smart dispensing cabinets; blood-product tracking systems	End-to-end provenance from manufacturer to patient; counterfeit detection; automated recall coordination via smart contracts	Moderate—case studies and supply-chain models (Chen et al., 2022; Sathiya et al., 2023)
EHR Sharing & Interoperability	Connected devices feed structured FHIR resources; HL7-compatible edge nodes normalise heterogeneous sensor formats	Hierarchical data-sharing with attribute-based access control; dual-blockchain separation of patient vs. provider chains; consent enforcement	Moderate—multiple prototypes and recent experimental systems; semantic interoperability remains standards-dependent (Zhang et al., 2022; Gohar et al., 2022; Othman & Getahun, 2025)
Clinical Trials & Research Governance	Remote monitoring devices replace site visits; decentralised trial architectures capture FHIR-structured endpoints	Immutable protocol version and consent records; tamper-evident deviation logs; contribution attribution for federated ML model training	Emerging-to-moderate—growing blockchain-federated learning literature; limited prospective clinical validation (Myrzashova et al., 2023; Wang et al., 2026)
Mental Health & Vulnerable Populations	Passive behavioural sensing (activity, sleep, voice biomarkers) with privacy-preserving edge processing before any data leaves the device	Patient-controlled selective disclosure; cryptographic consent delegation; ZKP-based proof of clinical eligibility without revealing diagnosis	Emerging, limited domain-specific validation; high sensitivity to consent, inference, discrimination, and re-identification risks (Khatri et al., 2023; Singh et al., 2021)

**Table 3:** Challenge Domains, Key Barriers, Mitigation Strategies, and Supporting Evidence.

Domain	Key Barriers	Mitigation Strategies	Supporting Evidence
Technical & Scalability	Consensus latency and throughput limits; resource constraints on low-power IoMT devices; legacy EHR/HIS integration; heterogeneous IoT protocols; 5G coverage gaps in rural settings	Permissioned consensus where justified; off-chain storage; batching/edge aggregation; FHIR-based interfaces and implementation guides; performance testing under realistic workload and failure conditions	Liu & Li, 2022; Myrzashova et al., 2023; Aboshosha et al., 2025; Zhang et al., 2025
Security & Privacy	Cryptographic key management burden; re-identification risk from on-chain metadata; ransomware targeting edge nodes; adversarial manipulation of IoMT sensor readings	HSM/secure-enclave key protection; recovery and revocation procedures; homomorphic encryption/ZKPs where proportionate; secure aggregation and differential privacy; network intrusion detection; device attestation and secure update	Ali et al., 2023; Alsemmeari et al., 2023; Kelli et al., 2021
Regulatory & Legal	GDPR data minimisation and erasure constraints; EHDS primary/secondary-use governance; AI Act and MDR/IVDR overlap for medical-device AI; cross-border roles and liability; NIS2 cybersecurity duties	Minimise personal data on-chain; document controller/processor roles; DPIA/risk management; standards-based interoperability; conformity-assessment alignment where applicable; incident-response and liability allocation	Ng et al., 2021; Mallick et al., 2024; Regulation (EU) 2025/327; Regulation (EU) 2024/1689 as amended; MDCG 2025-6; NIS2
Organisational & Cultural	Clinician resistance; institutional data-sovereignty concerns; siloed governance cultures; digital-literacy deficits among patients and staff	Co-design and participatory implementation; IHE profile adoption; national pilot programmes; change-management investment; progressive autonomy in patient consent UX	Adere, 2022; Rahman et al., 2022
Energy & Sustainability	Resource-intensive consensus mechanisms unsuitable for wearable/low-power devices; large-scale IoT network energy footprint	Energy-proportionate permissioned consensus; edge aggregation; workload-aware orchestration; measurement of device, network, storage, and consensus energy; renewable/efficient infrastructure where available	Liu & Li, 2022; Wendl et al., 2023 (clinical-scale whole-system evidence remains weak)
Economic & Equity	High infrastructure costs; uncertain ROI; digital divide between high- and low-income settings; risk of exacerbating health inequity	Total-cost-of-ownership analysis; phased deployment; open and standards-based procurement; accessibility and digital-inclusion measures; reimbursement/workforce planning; equity impact assessment	Mallick et al., 2024; WHO, 2021

**Table 4:** Evidence Strength Assessment for Key Claims.

Claim	Evidence Strength	Reasoning	Key Papers
Blockchain–IoMT integration can enhance provenance, tamper-evidence, and auditable access control when correctly architected	Moderate	Multiple prototypes and reviews support technical feasibility; security gains depend on endpoint integrity, key management, metadata exposure, and threat model	Zhang et al., 2022; Ali et al., 2023; Othman & Getahun, 2025; Shaikh et al., 2025
Patient-centric frameworks can support greater data agency, transparent access histories, and granular delegation	Moderate	Prototype systems demonstrate programmable access and delegation; legal rights, usability, recovery, and compliance remain context-dependent	Jabarulla & Lee, 2021; Gohar et al., 2022; Khatri et al., 2023; Rahman et al., 2024; Regulation (EU) 2025/327
Blockchain strengthens pharmaceutical supply chain resilience during pandemics	Moderate	Case studies and supply-chain models show improved traceability and transparency; limited live deployment evidence	Chen et al., 2022; Sathiya et al., 2023
5G/SDN-enabled IoMT can support low-latency, policy-aware remote monitoring under tested conditions	Moderate	Benchmarked prototypes support feasibility; large-scale clinical effectiveness and resilience under failure conditions remain insufficiently demonstrated	Rahman et al., 2024; Firouzi et al., 2021
Interoperability, scalability, endpoint trust, and governance integration remain major deployment barriers	Moderate	Reviews consistently identify heterogeneous standards, device/resource constraints, key management, and implementation complexity; few large-scale independent validations	Liu & Li, 2022; Myrzashova et al., 2023; Aboshosha et al., 2025; Zhang et al., 2025
Clinical adoption lags significantly behind technical development	Moderate	Recent systematic reviews remain dominated by architectures, simulations, prototypes, and benchmarks, with limited evidence of sustained clinical-scale benefit	Ng et al., 2021; Mallick et al., 2024; Shaikh et al., 2025; Wang et al., 2026
Energy and resource consumption are deployment considerations whose magnitude depends strongly on architecture and consensus	Weak	PoW is energy intensive; permissioned alternatives may reduce consensus energy, but whole-system clinical benchmarks remain sparse	Liu & Li, 2022; Wendl et al., 2023

Note: Evidence strength reflects maturity, realism, independent validation, and relevance to the stated claim; technical feasibility is not equivalent to demonstrated clinical effectiveness.

The evidence-strength categories in Table 4 follow an explicit three-tier qualitative rubric rather than a numerical score. A claim is rated Strong when findings are consistent across multiple independent research groups, evaluated under realistic or near-clinical conditions, and corroborated by at least one systematic review or meta-analysis. A claim is rated Moderate when it is supported by several independent prototypes, benchmarked architectures, or narrower systematic reviews, but the evidence remains confined to laboratory, simulation, or single-site testbed conditions, with limited independent replication or external validity. A claim is rated Weak when supporting evidence is limited to a small number of studies, proof-of-concept demonstrations, expert opinion, or extrapolation from adjacent domains, or when available studies report conflicting or inconclusive findings. Two members of the author team applied this rubric independently to each claim using the criteria described, and disagreements were resolved by discussion; this process, while not equivalent to formal GRADE or ROBIS appraisal, was intended to make the qualitative grading in Table 4 auditable and repeatable.

### 3.6. Use Case Analysis

#### 3.6.1. Remote Patient Monitoring and Telemedicine

The remote patient monitoring (RPM) domain has generated the densest body of blockchain–IoMT prototype evidence. Rahman et al. demonstrated an SDN-integrated patient-centric agent operating over 5G network slices, where smart contracts mediate access to IoMT streams and generate auditable delegation events; their prototype achieved sub-second latency under benchmarked conditions, supporting technical feasibility for real-time monitoring. Jabarulla and Lee proposed a blockchain–AI framework for COVID-19 patient monitoring that combines IoMT sensing with smart-contract-based consent management, while Othman and Getahun extended this to a full EHR-IoMT integration with dual-layer security. However, as Zhang et al. (2025) observe in their survey, most RPM prototypes report technical performance metrics (latency, throughput, transaction overhead) rather than clinical outcomes, and few have been validated with real patient populations over extended periods.

#### 3.6.2. Pandemic and Emergency Response

The COVID-19 pandemic catalysed numerous blockchain–IoMT proposals for outbreak surveillance and emergency response. Firouzi et al. positioned IoT, AI, robotics, and blockchain as complementary elements of a smart connected health ecosystem for pandemic management, while Ng et al. conducted a systematic review of blockchain applications in COVID-19 care, finding moderate evidence for contact tracing and data provenance but limited evidence for clinical effectiveness or population-level outcomes. The heterogeneity of deployment contexts, ranging from hospital load monitoring to syndromic surveillance, makes it difficult to generalise findings across settings. Moreover, the urgency of pandemic response often precluded the rigorous controlled evaluations that would be needed to establish comparative effectiveness.

#### 3.6.3. Pharmaceutical Supply Chain

Pharmaceutical and blood-product supply chains provide one of the more mature use cases for blockchain–IoMT integration, because provenance and chain-of-custody requirements map closely onto blockchain’s tamper-evident logging properties. Chen et al. describe how RFID/NFC tagging combined with distributed-ledger provenance can improve post-pandemic pharmaceutical supply-chain resilience by linking manufacturing, cold-chain, and dispensing events into a single auditable record. Sathiya et al. similarly model a chain-of-things architecture for healthcare supply chains, drawing explicit lessons from COVID-19-era vaccine and personal protective equipment shortages. Across these studies, the reported benefits are counterfeit detection, faster recall coordination, and improved cross-organisational visibility; however, the evidence remains largely limited to conceptual models, small pilots, and simulation, with few independently validated, large-scale deployments spanning manufacturer to point-of-dispensing. Cold-chain temperature loggers and smart dispensing cabinets illustrate a further integration point with Layer 1 of the proposed framework, but their clinical value depends on integration with existing enterprise resource-planning and pharmacy-inventory systems rather than on blockchain infrastructure alone.

#### 3.6.4. Clinical Trials and Research Governance

Blockchain–IoMT integration for clinical trials and research governance remains an emerging rather than an established use case. The proposed value lies in immutable protocol-version and consent records, tamper-evident deviation logging, and contribution attribution for federated model training across trial sites, extending the patient-centric consent mechanisms described in Section 3.4 to a research context. Myrzashova et al.’s systematic review of blockchain-federated learning in healthcare identifies decentralized clinical-trial monitoring as a promising application area, while the remote-monitoring devices reviewed could in principle replace some site visits and capture FHIR-structured endpoints directly from participants. However, at the time of this update no peer-reviewed study had reported a prospective, blockchain-governed clinical trial with patient-centred outcomes; the literature remains dominated by architectural proposals and simulation-based federated-learning benchmarks, such as those from Murthy and Shri, and Bhasker et al. Regulatory considerations specific to this domain include Good Clinical Practice data-integrity requirements and the EHDS secondary-use provisions, neither of which is satisfied merely by ledger-based logging.

#### 3.6.5. Mental Health and Vulnerable Populations

Mental health monitoring presents distinct governance challenges due to the inferential nature of passive behavioural sensing (activity patterns, sleep, voice biomarkers). Khatri et al. proposed a Hyperledger Fabric-based framework for patient-centric mental health data management, incorporating cryptographic consent delegation and ZKP-based clinical eligibility proofs. Singh et al. similarly addressed vulnerable populations with a patient-centric architectural framework that emphasizes selective disclosure and consent granularity. However, the sensitivity of mental health data to re-identification and secondary inference remains under-addressed in the blockchain literature: on-chain metadata such as transaction timing or interaction patterns can reveal psychological status even when clinical content is encrypted off-chain. The evidence base for blockchain–IoMT in mental health settings remains limited to prototype and simulation studies, with no prospective clinical validation in peer-reviewed literature through August 2026.

## 4. Discussion

### 4.1. Blockchain as a Trust Layer, Not a Database

A foundational principle of the framework is that blockchain should ordinarily function as a governance, coordination, and verification layer rather than as the primary repository for clinical content. Continuous IoMT telemetry, EHR documents, genomic data, and imaging are poorly matched to replicated on-chain storage because of volume, latency, confidentiality, retention, and cost constraints. Hybrid architectures therefore retain clinical data off-chain and anchor selected hashes, pointers, access events, consent-policy state, and provenance metadata to the ledger [11,19,20]. This separation also allows personal data to remain within storage environments where lifecycle controls can be applied more directly.

Blockchain can strengthen three properties when appropriately designed: integrity verification through cryptographic anchoring; shared, tamper-evident audit histories across organizational boundaries; and deterministic execution of defined governance rules through smart contracts. None are exclusive to blockchain, and each has limits. A hash proves consistency with an anchored state but not the clinical truth of the source measurement; an append-only audit log is useful only if identities, clocks, devices, and event capture are trustworthy; and executable consent logic cannot replace legal-basis assessment, emergency-access governance, or human oversight. Multi-ledger separation may improve compartmentalization but also increases governance and recovery complexity [16,18].

A more granular threat model further clarifies the boundaries of blockchain’s contribution to security. Distinct adversarial scenarios include: compromised or spoofed IoMT endpoints that inject falsified telemetry before it ever reaches the ledger, against which blockchain offers no inherent defence; malicious or colluding validator nodes within a permissioned consortium, which threaten consensus integrity and require robust node-admission and governance controls rather than cryptography alone; denial-of-service attacks against gateways, edge nodes, or the consensus network, which can degrade availability of latency-sensitive monitoring without breaching confidentiality; smart-contract logic errors or exploits that can misallocate access rights or trigger unintended state transitions; and, in federated-learning-integrated architectures, model-poisoning and gradient-inversion attacks that can corrupt or de-anonymise training even when the ledger correctly records model-version provenance [23,43]. Machine-learning-based intrusion detection is a recognised complementary control for the endpoint- and network-layer components of this threat model: signature- and anomaly-based approaches have been used to detect intrusions in resource-constrained and distributed networks structurally similar to IoMT deployments, and dedicated benchmark datasets have been developed to support standardised evaluation of such systems [47,48,49]. Realistic evaluation of blockchain–IoMT security therefore requires threat-model-specific testing (endpoint attestation, consensus fault injection, adversarial federated-learning simulation) rather than reliance on the cryptographic properties of the ledger alone, reinforcing the evidence gap identified as the third priority research question.

### 4.2. The 5G/SDN-IoMT Continuum: From Sensing to Accountable Data

The combination of IoMT, programmable networking, edge services, and blockchain can create an end-to-end accountability chain from sensing to authorized use. In the Rahman et al. prototype, an SDN control plane coordinates 5G-connected monitoring while a patient-centric agent applies blockchain-encoded access policies and records delegation events [9]. The broader design principle is network-agnostic: governance should remain explicit whether transport is 5G, Wi-Fi, wired hospital infrastructure, or another secure channel, and latency-critical safety functions should not depend unnecessarily on remote consensus.

This architecture may strengthen adverse-event reconstruction, pharmacovigilance, and accountability by preserving timestamped provenance for device states, access rights, software versions, and supply-chain events. It should not be described as automatically producing legally conclusive evidence: evidentiary value still depends on secure device identity, trusted time sources, firmware integrity, chain-of-custody procedures, and the accuracy of the originating sensor or human input. Blockchain can preserve a false or manipulated input just as reliably as a correct one. Linking trusted RFID/NFC supply-chain events to clinical data may nevertheless improve traceability from manufacturing batch to downstream use [50,51].

At population level, Firouzi et al. position IoT, AI, robotics, and blockchain as complementary elements of a smart and connected health ecosystem [4]. The relevant implication for governance is modularity: each technology should be selected for a defined function and evaluated against simpler alternatives, rather than assuming that blockchain or 5G is intrinsically necessary for every digital-health workflow.

### 4.3. Privacy-Preserving Computation: Extending Governance into Analytics

Privacy-enhancing computation should be treated as a first-class governance concern because no single mechanism resolves the privacy-utility trade-off. Homomorphic encryption can reduce plaintext exposure during defined computations; ZKPs can support selective proofs; federated learning can keep raw training data at institutional nodes; secure aggregation and differential privacy can further limit disclosure from model updates [22,23,24,43]. Each technique introduces distinct assumptions and costs. Federated learning is not synonymous with privacy, and cryptographic protection of computation does not address purpose limitation, data quality, representativeness, or inappropriate downstream use.

The value of combining these techniques lies in separation of responsibilities: the ledger can record policy state, provenance, model versions, and contributions; cryptographic mechanisms can protect selected computations or proofs; and federated learning can distribute training. HealthLock provides an IoT-healthcare example of homomorphic encryption [22], while systematic reviews published in 2023 and 2026 show rapid growth in blockchain-federated learning research but persistent limitations in scalability, attack resistance, privacy leakage, interoperability, and real-world validation [23,43]. The 2026 tutorial literature further emphasizes that regulatory compliance must be engineered across the full data and model lifecycle rather than inferred from the presence of a privacy technology [24]. Recent architectures extend this integration by combining blockchain-governed federated IoMT with reinforcement-learning-based consensus and resource forecasting, and by coupling blockchain, IoT devices, and federated learning in sustainability-oriented healthcare systems [52,53].

### 4.4. Patient-Centricity: From Policy to Operational Reality

The literature increasingly operationalizes patient-centered governance in prototypes that expose consent, access histories, and delegation controls to patients [18,36,37,44]. The main translation gap remains movement from laboratory, simulation, or limited testbed evidence to sustained deployment within clinical workflows [2,16,17]. Newer systems such as EHRGuard add experimental evidence for integrated blockchain–IoMT EHR architectures [11], but this should not be conflated with proof of improved patient outcomes, safety, cost-effectiveness, or acceptability at health-system scale.

Three implementation issues are recurrent. First, key and identity lifecycle management must support recovery, delegation, revocation, incapacity, proxy access, and emergency care without shifting an unrealistic security burden to patients. Second, the relationship between immutable ledger records and data-protection obligations requires data minimization, careful treatment of pseudonymous metadata, off-chain storage of mutable personal data where appropriate, and explicit controller/processor role allocation rather than a simple technical “right-to-erasure solution”. Third, decentralized architectures distribute responsibility across device manufacturers, software providers, healthcare organizations, infrastructure operators, and users, making liability, incident response, and governance escalation central design requirements [16,46].

Beyond consent and identity management, organisational adoption barriers deserve explicit treatment because they are frequently the binding constraint on deployment rather than the underlying cryptography. Clinician-facing integration typically requires blockchain-mediated access and consent events to surface within existing electronic health record and clinical-decision-support interfaces rather than through a separate application, since additional login steps or parallel dashboards are a recognised driver of workflow friction and alert fatigue. Table 3 (Organisational & Cultural domain) summarises the associated barriers, clinician resistance, siloed governance cultures, and digital-literacy gaps, and corresponding mitigations such as co-design, IHE profile adoption, and change-management investment; the evidence base for these mitigations, however, consists largely of implementation-science commentary and small pilot deployments rather than controlled organisational studies, and should be interpreted accordingly.

### 4.5. Regulatory Convergence: EHDS, AI Act, Medical Devices, and Cybersecurity

For European deployments, blockchain–IoMT governance now sits within a denser regulatory architecture than the earlier literature generally assumed. Regulation (EU) 2025/327 establishing the European Health Data Space introduces common rules, standards, infrastructures, and governance for primary and secondary use of electronic health data [29]. This strengthens the case for standards-based data portability, traceable access, and accountable secondary use, but it does not privilege blockchain as the required implementation mechanism. The architectural implication is that ledger functions should remain interoperable with EHDS data-access and governance processes and should minimize the placement of personal data on immutable infrastructure.

AI-enabled components introduce a second layer of obligations. Regulation (EU) 2024/1689 (AI Act), as amended in 2026, operates alongside the Medical Devices Regulation and In Vitro Diagnostic Medical Devices Regulation when AI is incorporated into regulated medical products [30,31,32]. As of 10 August 2026, the amended timetable applies the high-risk AI requirements for AI systems embedded in products covered by the Union harmonization legislation listed in AI Act Annex I from 2 August 2028 [31]. The practical governance requirements include risk management, data governance, technical documentation, logging and traceability, human oversight, cybersecurity, and post-market responsibilities. Blockchain may support selected logging, provenance, and accountability functions, but it cannot substitute for conformity assessment or lifecycle risk management. At the organizational level, NIS2 adds cybersecurity governance and incident-management obligations for many health-sector entities [33]. A deployable architecture must therefore map each technical control to a specific legal actor, purpose, risk, and evidence requirement.

Beyond the high-level obligations, specific regulatory timelines and implementation requirements shape the practical design of blockchain–IoMT architectures. Under the EHDS, Member States are required to establish national health data access bodies and interoperable infrastructures for secondary use of electronic health data by 2027–2028; the implementing acts specifying technical standards are under development as of August 2026. For blockchain–IoMT deployments, this implies that ledger functions, such as consent-state recording, access logging, and provenance anchoring, must be capable of interoperating with EHDS data-access portals and the MyHealth@EU infrastructure for cross-border data exchange. The EHDS does not mandate blockchain as an implementation mechanism; rather, it requires auditable access, purpose limitation, and patient transparency, all of which can be supported by blockchain but may also be achieved through conventional audit systems.

For AI-enabled components, the interplay between the AI Act and the medical device regulations has been clarified by the joint AIB 2025-1/MDCG 2025-6 guidance. The guidance establishes that AI systems incorporated into medical devices classified under MDR/IVDR are subject to both regulatory frameworks, with conformity assessment pathways determined by the device risk class and the AI system’s intended use. The 2026 amendment to the AI Act extends the transitional period for certain high-risk AI systems, with the requirements for AI systems embedded in products covered by Annex I harmonization legislation applying from 2 August 2028. For blockchain–IoMT systems that incorporate machine learning for clinical decision support, this means that developers must prepare technical documentation, risk management files, and post-market monitoring plans that satisfy both the AI Act and the applicable device regulation; blockchain may support logging and traceability but cannot substitute for these substantive conformity obligations.

The NIS2 Directive, transposed into national law by Member States, imposes cybersecurity risk-management and incident-reporting obligations on health-sector entities. For IoMT devices and blockchain-based governance layers, this translates into requirements for secure key storage, access controls, supply-chain risk assessment, and incident notification within 24–72 h of detection. Recent national implementations, such as Ireland’s Health Information Act 2026, provide examples of how NIS2 obligations are being operationalized in health settings. A deployable blockchain–IoMT architecture must therefore map each technical control to a specific legal actor, purpose, risk, and reporting pathway, rather than asserting general compliance through technology selection alone.

### 4.6. Evidence Gaps and Future Research

The updated evidence map shows a consistent maturity gradient: cryptographic access control, provenance, and prototype performance are relatively well represented, whereas prospective clinical evaluation, independent security testing, workflow integration, economic impact, equity, and long-term sustainability remain under-evidenced [11,16,17,23]. Interoperability claims also require greater precision. Blockchain can coordinate identities, policies, and provenance, but semantic interoperability depends on standards such as HL7 FHIR, terminologies, implementation guides, mapping quality, and organizational governance [54].

A related and frequently underexplored question is how blockchain-governance layers interoperate with the substantial installed base of legacy hospital information systems, many of which still rely on HL7 v2 messaging, proprietary interfaces, or point-to-point integration engines rather than native FHIR APIs. In these environments, blockchain-based consent and access-control logic typically cannot act directly on clinical data held in legacy systems; it instead requires an intermediary integration layer (e.g., an HL7 v2-to-FHIR gateway or enterprise service bus) capable of translating access decisions into calls the legacy system can enforce. This intermediary layer, rather than the ledger itself, often determines whether the proposed governance model is practically deployable, and its cost and complexity are rarely reported in the prototype literature reviewed here. Comparative implementation studies that quantify integration effort against legacy infrastructure, rather than against greenfield FHIR-native testbeds, represent a further priority alongside the five research questions above.

A further scope boundary worth noting explicitly is medical imaging, a long-standing and comparatively mature adjacent application area for blockchain in healthcare that falls outside the six use-case domains examined in Section 3.6. Prior work has proposed blockchain-based provenance and access-control mechanisms for medical images and has begun to integrate these with FHIR- and DICOMweb-based imaging workflows [55,56,57]. The governance principles developed here, minimisation of on-chain personal data, off-chain storage of large clinical objects, and ledger-anchored provenance, are broadly transferable to that domain, but a dedicated review would be needed to assess the imaging-specific evidence base.

Synthesizing the evidence gaps identified across the reviewed literature, five priority research questions emerge for the 2026–2031 period:

First, which governance problems genuinely require a distributed ledger rather than a conventional trusted service? The literature consistently shows that blockchain adds value for provenance, multi-organizational audit, and programmable consent, but simpler alternatives, such as databases with cryptographic hashing and conventional access logs, may be sufficient for intra-organizational use cases. Comparative implementation studies are needed to identify the specific conditions (e.g., number of participating organizations, regulatory stringency, trust asymmetries) under which blockchain’s overhead is justified.

Second, how can interoperable identity, consent, provenance, and FHIR-based data exchange be benchmarked across platforms? Recent surveys identify interoperability as a persistent barrier, yet there is no standardized benchmarking framework for blockchain–IoMT systems. The development of open testing environments with representative clinical workloads, device heterogeneity, and failure scenarios would enable meaningful comparisons.

Third, how do systems behave under realistic threat models, including compromised sensors, stolen credentials, malicious federation participants, metadata inference, and network partitions? Most prototype evaluations assume benign conditions and do not test adversarial scenarios. Independent security testing and red-team exercises are needed to move beyond architecture-level security claims.

Fourth, can privacy-enhancing and energy-efficient designs remain performant at clinical scale? Recent work on reinforcement-learning-based resource forecasting and adaptive consensus suggests promising directions [58], but these have been validated primarily in simulation. Prospective evaluations in clinical settings with actual patient populations are required.

Fifth, does implementation improve safety, access, trust, cost, or health outcomes without widening digital inequities? The current evidence base is dominated by technical feasibility studies; the translation to patient-relevant outcomes remains the central implementation challenge. Equity impact assessments, digital inclusion measures, and cost-effectiveness analyses are notably absent from the literature.

### 4.7. Industry 5.0 Alignment

The Industry 5.0 paradigm provides a useful ethical and design lens through its emphasis on human-centricity, sustainability, and resilience. Within the five-layer framework, these principles translate into specific architectural choices. Human-centricity requires that patient-facing interfaces for consent delegation, access history review, and emergency authorization are usable by diverse populations, including those with limited digital literacy; recent work on patient-centric frameworks has begun to address these requirements but has not been systematically evaluated in real-world populations. Sustainability favours energy-proportionate permissioned consensus and edge aggregation over resource-intensive Proof-of-Work designs; Liu and Li’s analysis of energy-efficient IoMT architectures provides a baseline for this dimension, though whole-system energy benchmarks remain sparse [38,59]. Resilience requires redundancy, offline-safe clinical modes, tested recovery procedures, and avoidance of new single points of failure in identity providers, gateways, key-management services, or consensus membership. Emergency access should be governed by predefined, auditable break-glass policies rather than an unspecified relaxation of privacy protections, as emphasized in the patient-centric agency literature [26].

### 4.8. Limitations

This review has important limitations. It is a focused narrative review rather than a prospectively registered systematic review: the protocol was not registered, there was no fixed PICO-type question, and full-text screening and evidence grading were not performed independently by two reviewers throughout; study selection and qualitative evidence grading therefore remain vulnerable to selection and interpretation bias, and no pooled estimates of performance, cost, safety, or clinical effect are provided. These limitations are intrinsic to the narrative review design and are partly mitigated, but not removed, by the transparent multi-database search and the explicit evidence-grading rubric. The framework is conceptual and has not been prospectively validated as an integrated architecture. Consistent with this conceptual status, the review does not provide a quantitative, head-to-head comparison of blockchain platforms, consensus mechanisms, or IoMT security architectures on common workloads, nor does it include an experimental implementation, simulation, or structured expert-elicitation exercise to validate the five-layer model itself; such validation is identified as a priority and is left to dedicated future work. The evidence base remains dominated by prototypes, simulations, surveys, and architecture papers, with substantial heterogeneity in metrics and threat models. The targeted 2026 update was designed to capture major recent peer-reviewed and authoritative regulatory developments rather than to constitute a new exhaustive systematic search. Finally, platform capabilities, standards, EU implementation timelines, and AI/medical-device guidance continue to evolve; deployment decisions should therefore use the current versions of applicable standards and law.

## 5. Conclusions

Blockchain and IoMT can be complementary components of digital-health governance, but neither is sufficient on its own and neither should be adopted as an end. The updated synthesis supports a more qualified conclusion than technological advocacy: distributed ledgers can add value where multiple parties need shared provenance, auditable policy execution, and coordinated trust, while IoMT supplies the sensing infrastructure for continuous and distributed care. The strength of evidence is currently greatest for technical feasibility and architecture-level security properties, and weaker for clinical effectiveness, cost-effectiveness, equity, and sustained health-system deployment [11,16,17,23].

The proposed five-layer model—IoMT acquisition; network and edge orchestration; blockchain governance; privacy-enhancing data and AI services; and application delivery—is intended as a modular decision framework rather than a prescriptive technology stack. Its contribution is to make the interfaces between sensing, connectivity, governance, computation, and service delivery explicit; to position patient-centric delegation and privacy-enhancing computation within those interfaces; and to overlay the architecture with calibrated evidence and contemporary European governance requirements, including EHDS, the AI Act/MDR-IVDR interplay, and NIS2 [29,30,31,32,33].

The next step is not broader deployment by default, but disciplined translation. The path from technically credible prototypes to demonstrably safe, effective, equitable, and sustainable clinical systems requires a shift in the evaluation standards applied to blockchain–IoMT research.

To address this gap, future implementations should:

1. Begin with a defined governance problem and explicit threat model, demonstrating that a distributed ledger adds measurable value over simpler alternatives rather than being adopted as a technological default.
2. Use standards-based interoperability (HL7 FHIR, IHE profiles, GS1 identification standards) as the foundation for semantic integration, with blockchain serving as a governance overlay rather than a data transport mechanism.
3. Minimize on-chain personal data through careful schema design, off-chain storage of clinical content, and differential privacy or secure aggregation for ledger metadata where appropriate.
4. Undergo independent security, usability, regulatory, and clinical evaluation, including red-team testing, user acceptance studies, and prospective clinical validation with patient-centred outcomes.
5. Address equity and digital inclusion through accessibility measures, multilingual interfaces, low-bandwidth modes, and affordability assessments, as emphasized in recent equity-oriented health technology assessments [46,60].

The research and implementation community should therefore prioritize rigorous translation over broad deployment by default. The present framework offers a modular decision aid for that translation, but its utility will ultimately be determined by the quality of the evidence generated in real-world deployments over the coming years.

## 6. COPE Statement

Generative AI tools were used for language editing and structural refinement. All source verification, scientific interpretation, and final decisions remain the responsibility of the authors.

## Data Availability

Dataset available on request from the authors.

## Abbreviations

Internet of Things	IoT	Fifth-Generation Mobile Network	5G
InterPlanetary File System	IPFS	Artificial Intelligence	AI
Machine Learning	ML	Blockchain as a Service	BaaS
Near-Field Communication	NFC	Coronavirus Disease 2019	COVID-19
Artificial Intelligence Act	AIA	Decentralised Identifier	DID
European Health Data Space	EHDS	Electronic Health Record	EHR
Proof of Authority	PoA	Electronic Medical Record	EMR
Proof of Stake	PoS	Fast Healthcare Interoperability Resources	FHIR
Radio-Frequency Identification	RFID	General Data Protection Regulation	GDPR
Return on Investment	ROI	Graphics Processing Unit	GPU
Remote Patient Monitoring	RPM	Healthcare Cyber-Physical Systems	HCPS
Software-Defined Networking	SDN	Hospital Information System	HIS
Federated Learning	FL	Hardware Security Module	HSM
World Health Organization	WHO	Integrating the Healthcare Enterprise	IHE
Zero-Knowledge Proof	ZKP	Internet of Medical Things	IoMT
		Network and Information Systems Directive 2	NIS2
